# Surgical treatment of neuromuscular Early Onset Scoliosis with a bilateral posterior one-way rod compared to the Spring Distraction System: study protocol for a limited-efficacy Randomized Controlled Trial (BiPOWR)

**DOI:** 10.1186/s12891-022-06048-4

**Published:** 2023-01-10

**Authors:** Justin V.C. Lemans, Casper S. Tabeling, E. Pauline Scholten, Hilde W. Stempels, Lotfi Miladi, René M. Castelein, Moyo C. Kruyt

**Affiliations:** 1 Department of Orthopaedic Surgery, University Medical Center Utrecht, P.O. Box 85500, 3508 GA Utrecht, The Netherlands; 2 Hôpital Necker-Enfants Malades, Paris, France; 3 Twente University, Enschede, The Netherlands

**Keywords:** BiPOWR, Early Onset Scoliosis, Neuromuscular, Growth, Spring Distraction System, SDS, One Way Self-Expanding Rod, OWSER, Pediatric spine, Bipolar technique

## Abstract

**Background:**

Early Onset Scoliosis (EOS) is a progressive spinal deformity in children, and a potentially life-threatening disease. “Growth-friendly” surgical techniques aim to control the deformity, while allowing the spine and trunk to maintain growth. Current “growth-friendly” systems such as the traditional growing rod (TGR) and magnetically controlled growing rod (MCGR) have limitations that reduce their efficacy and cost-effectiveness. Recently, two “growth-friendly” systems have been developed that mitigate many of these limitations, the Spring Distraction System (SDS) and the One Way Self-Expanding Rod (OWSER). The purpose of the multicenter BiPOWR trial is to investigate, describe and compare the 1-year limited-efficacy and -safety of both strategies in the treatment of neuromuscular EOS.

**Methods:**

After informed consent, 28 neuromuscular EOS patients will be randomized to receive either the SDS or the OWSER. Patients and caregivers will be blinded to allocation until after surgery. Primary outcomes will be maintenance of coronal curve correction and the occurrence of serious adverse events. In addition, spinal growth, implant lengthening, and perioperative findings are recorded systematically. At each follow-up moment, the Early Onset Scoliosis Questionnaire (EOSQ-24) will be used to assess health-related quality of life. All outcomes will be compared between groups.

**Discussion:**

The BiPOWR trial is the first randomized controlled trial that compares two specific “growth-friendly” implants in a specified EOS population. It will determine the 1-year limited-efficacy and safety of the SDS and OWSER implants.

**Trial registration:**

Clinicaltrials.gov: NCT04021784 (13–06-2019). CCMO registry: NL64018.041.17 (06–05-2019).

## Background

Early Onset Scoliosis (EOS) is a complex three-dimensional deformity of the spine and trunk, which, if left untreated, can result in severe cardiopulmonary compromise or even death [1, 2]. The early onset of the deformity carries with it a high risk of progression, which places importance on early treatment to preserve pulmonary function. This is even more important in patients with neuromuscular diseases such as spinal muscular atrophy or Duchenne muscular dystrophy. In these patients, respiratory muscles that are necessary for normal in- and expiration weaken, which, together with altered chest wall compliance, leads to further respiratory compromise [3, 4]. Whilst a spinal fusion is able to correct the deformity, doing this in the growing spine arrests all further growth, which limits pulmonary function even more [5]. In addition, continued anterior spinal growth after posterior fusion may result in worsening of the curve, a phenomenon known as crankshafting [6]. To circumvent these problems, “growth-friendly” techniques have been developed, which control curve progression while allowing for the spine and trunk to grow. Currently, the most common “growth-friendly” systems are the traditional growing rod (TGR) and the magnetically controlled growing rod (MCGR) [7, 8]. While both systems can control the scoliotic deformity, they have important limitations. For the TGR, the most obvious limitation is the need for repetitive surgical lengthenings (generally every 6 months), which exposes patients to high anesthetic stress at a young age, which may negatively influence neurodevelopment [9]. For the MCGR, lengthenings are non-invasive, but they still require patients to come to the outpatient clinic on a regular basis, more often than in TGR, which imposes a psychological burden on these patients and their parents [10]. In addition, both TGR and MCGR have a high mechanical (i.e. implant-related) complication rate which results in reoperations and diminished length gain [11–16]. The high rate of MCGR dysfunction recently resulted in a temporary suspension of CE certification and the advice to limit MCGR implantation in several countries [17, 18]. To overcome these limitations, two new systems were developed: the Spring Distraction System (SDS), which uses a compressed spring around standard rods to generate distraction, and the One Way Self-Expanding Rod (OWSER, CE-marked as Nemost®; Euros, SAS, La Ciotat, France), which is a one-way sliding rod with split retaining ring system. Both systems are designed to facilitate growth of the spine without interventions, while maintaining curve correction. Previous publications have shown the feasibility of both systems [19, 20]. However, patient populations and surgical strategies were heterogeneous, and the case series design could have led to selection bias. To determine clinical efficacy, identify early mechanical failures, and to generate level 1 evidence for potential differences between the techniques, a single-blinded, prospective randomized trial was considered the ideal strategy.

## Methods

### Study aims

The primary aim of the BiPOWR trial is to prospectively describe and compare efficacy (coronal and sagittal curve maintenance) and –safety (occurrence of Serious Adverse Events; SAEs) of two innovative implant systems in neuromuscular EOS patients. Due to the relatively short follow-up of participants (1 year), with intermediate instead of final outcomes, the study is regarded as a limited-efficacy and -safety study [21]. A secondary aim is to describe and compare spinal- and implant growth, peri-operative parameters and Health-Related Quality of Life (HRQoL).

### Study design

The BiPOWR trial is a prospective, multicenter, randomized, surgical trial with two parallel groups and a 1:1 allocation ratio. Ethical approval was provided by the Institutional Review Board of the UMC Utrecht (METC 18–179). Following inclusion, patients undergo “growth-friendly” scoliosis surgery with one of two implants, the SDS or the OWSER. Which implant they receive is decided by randomization. Following implantation, the patients are followed until 12 months post-operatively, during which their radiographic- and clinical outcomes will be described and compared. The analysis at 12 months provides a point for interim analysis in the short follow-up, to determine whether one or both implants show short-term technical malfunctions, and to ascertain whether early detectable differences are present in other outcome domains. After the 1 year analysis, both patient cohorts are followed bi-yearly for several more years, until after skeletal maturity, to show efficacy and safety in the long-term. A SPIRIT table showing a detailed follow-up timeline for the first year of follow-up is shown in Table 1.

**Table 1:** SPIRIT table

	**STUDY PERIOD**							
	**Enrolment**	**Allocation**	**Surgery**	**Post-allocation**				
TIMEPOINT**	***-t***_***2***_ Before -2 weeks	***-t***_***1***_ -2 weeks	***t***_***0***_ Day of surgery	***t***_***1***_ + 1 week	***t***_***2***_ + 4 weeks	***t***_***3***_ 3 months	***t***_***4***_ 6 months	***t***_***5***_ 12 months
**Enrolment**								
Eligibility screen	X							
Informed consent	X							
Allocation		X						
**Interventions**								
Spring Distraction System			**Implant in Situ**	**Implant in Situ**	**Implant in Situ**	**Implant in Situ**	**Implant in Situ**	**Implant in Situ**
One Way Self-Expanding Rod			**Implant in Situ**	**Implant in Situ**	**Implant in Situ**	**Implant in Situ**	**Implant in Situ**	**Implant in Situ**
**Outcomes**								
Main coronal Cobb angle	X			X	X	X	X	X
Serious Adverse Events			X	X	X	X	X	X
Implant length				X	X	X	X	X
EOSQ-24 questionnaire	X			X	X	X	X	X

### Eligibility criteria

Inclusion criteria are:

- Neuromuscular or syndromic EOS (i.e. diagnosis before age 10).
- Progressive EOS with an indication for bipolar fixation extending to the pelvis.
- Non-ambulant patients.
- Age < 12 years.

Exclusion criteria are:

- Closed triradiate cartilage.
- Main curve proximal end vertebra at or above T3.
- Presence of skeletal dysplasia affecting growth.
- Presence of disease that severely influences bone quality or is associated with soft tissue weakness.
- Presence of active systemic disease (other than neuromuscular disease).
- Congenital spinal anomaly of  > 5 vertebrae.
- Previous instrumented spinal surgery.
- Patients who cannot be followed for 1 year post-operatively.

### Recruitment and informed consent

The treating physician identifies potential eligible patients, and asks the patient and their caregiver(s) whether they would like to receive additional study information. Upon agreement, the principal investigator is consulted for approval and the research team subsequently sends the study information. After at least two weeks, a consultation with the research team takes place where the in- and exclusion criteria are assessed, the study is explained in detail and patients’ questions are answered. Care is taken to ensure that the study design (in particular blinding and randomization) and the similarities and differences of the two treatment arms are understood. If willing to participate, the informed consent form is signed in duplicate and the consent is noted in the electronic patient record.

### Randomization and blinding

After inclusion, the patient is randomized into one of two treatment groups. Before the start of the trial, a randomization sequence was created using a computer-generated random number sequence, which was used to create a permuted block design with random block sizes. A random number of blocks with random block sizes of 2, 4, 6 and 8 created the final sequence containing 28 randomized patients. The sequence was converted into allocation notes, which were stored in opaque, sealed envelopes. Each allocation envelope was sealed into a second, sequentially numbered, opaque envelope. The use of two envelopes prevents the use of trans-illumination to unblind allocation. Two weeks before surgery, the next numbered study envelope is retrieved by two members of the study team. The envelopes, the seals in particular, are visibly examined for tampering. The allocation is confirmed by both research team members, and the pseudonymized patient number is written on the allocation note and the study envelopes. The treating physician is then notified of the outcome of the allocation, to order the required implants.

The patient and caregiver(s) remain blinded to the result of allocation until after surgery to prevent selection bias through termination from the study before surgery commences, which is a possibility if they have a strong preference for one treatment arm [22]. After surgery has taken place, the allocation outcome is shared with the patient and caregiver(s). While blinding patients during the entire study period is theoretically possible, enforcing this would require extreme effort, as it would preclude the treating physician of showing any of the follow-up radiographs to the patient and caregiver(s) as these clearly show the different implants. Since these radiographs often provide a wealth of information to the patient and caregiver(s), denying them these raises ethical concerns and would likely decrease enrolment rate.

### Surgical procedure and treatment arms

All surgical procedures will be performed using a less invasive bipolar posterior approach with somatosensory and motor-evoked potential monitoring [23]. The distal anchor is created with iliosacral screws (Tanit®; Euros, SAS, La Ciotat, France). The proximal anchor for SDS consists of bilateral pedicle screws at 3 consecutive levels, typically T2-T4. For the OWSER, a series of laminar- and pedicle hooks spanning 5 vertebrae is applied (T2-T6).

**Fig. 1:**
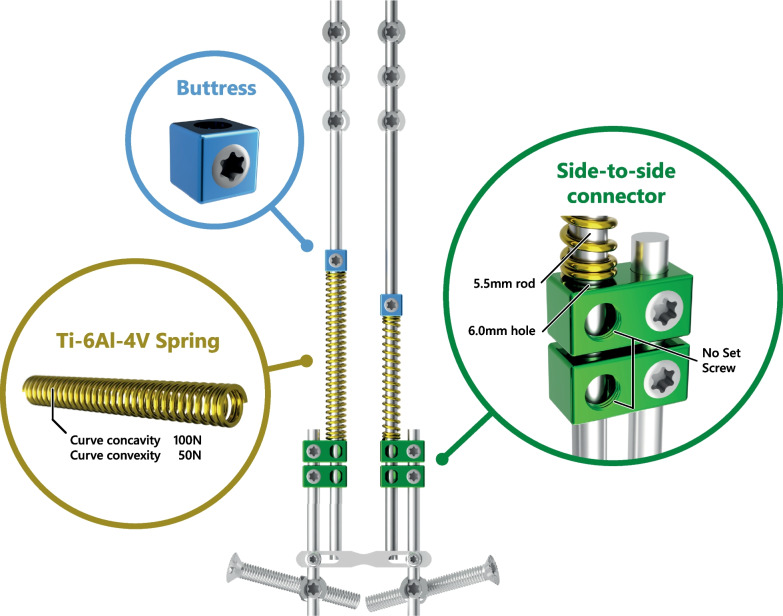
Spring Distraction System. The SDS consists of three components that are added to standard growing rods. It provides a continuous distraction force during follow-up, without the need for repeated lengthenings. In the BiPOWR trial, the distal anchors are iliosacral screws instead of pedicle screws. Green: A side-to-side connector with one oversized hole through which a CoCr rod can slide freely. Gold: Ti6Al4V springs which can be compressed over the rod. Blue: The buttress compresses the spring against the side-to-side connector

The SDS (Fig. 1) is an adjunct to standard 5.5 mm cobalt-chromium (CoCr) rods. It consists of three components: (1) a titanium (Ti6Al4V) spring placed over the rod, (2) two stacked oversized parallel connectors and (3) a buttress used to tension the springs (Stryker, Leesburg, VI, USA). In neuromuscular EOS, the SDS is placed bilaterally. The concave rod receives a strong 100 N spring (spring constant (*k*) = 1.32 N/mm), the convex rod receives a weaker 50 N spring (*k* = 0.68 N/mm). Around 6–7 cm of residual rod length is left for growth. The parallel connectors are secured to the distal anchor rods and the rod containing the spring is allowed to slide in the connector.

**Fig. 2:**
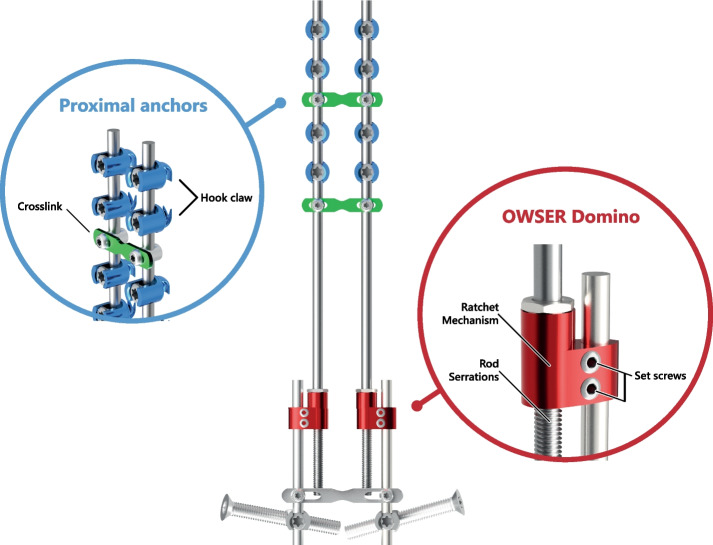
One Way Self-Expanding Rod. The OWSER is a growing rod that passively lengthens one way as the spine grows. In the BiPOWR trial, the distal anchors are iliosacral screws instead of pedicle screws. Blue: The proximal fixation consists of hooks positioned in a claw configuration. Two crosslinks are added for torsional stability (green). Red: The growing domino, combined with a rod that is serrated across its distal length, allows for lengthening. The reserve length can be 50 mm or 80 mm long. Movement in the other direction is prevented by a split retaining ring system inside the domino

The OWSER (Fig. 2) consists of two titanium components: (1) a 5.5 mm titanium long rod with a notched end, and (2) a sliding domino that passively migrates only one way across the notched rod segment. The long rod is connected to the proximal anchor with the notched end facing distally. The connector is fixated with a short rod to the distal anchor. Implant growth potential (i.e. the notched segment of the long rod) can be 50 or 80 mm. In neuromuscular EOS, the OWSER is also placed bilaterally.

**Fig. 3:**
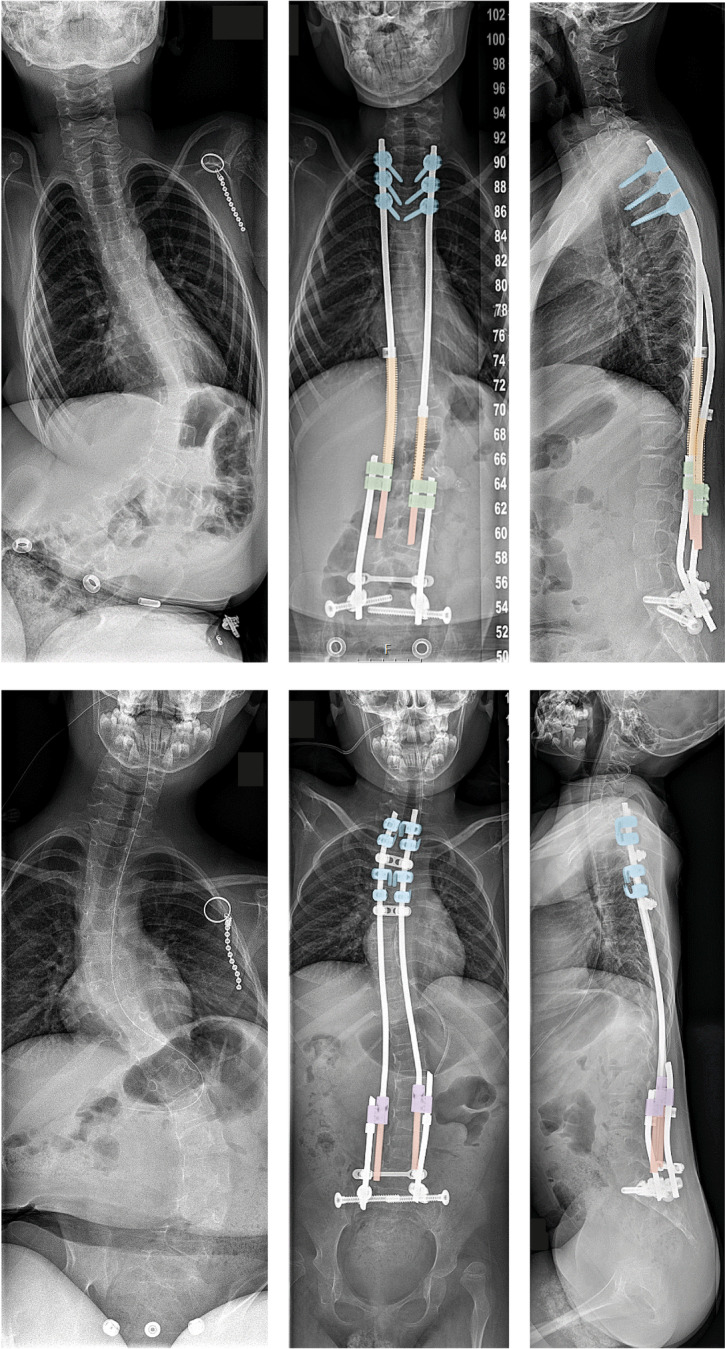
Implant configuration. Representative implant configurations of the SDS (top) and the OWSER (bottom) are shown. Top. A proximal foundation of 3 pedicle screws is placed (blue). The distal foundation consists of iliosacral screws. CoCr rods are placed through open side-to side connectors (green). Around these rods, 2 springs (orange) are positioned, which push against these connectors and a proximal locking buttress. The gliding parts of the rods (red) become shorter as the spine grows. Bottom: Here, the proximal foundation consists of 2 claws (blue), created with hooks. The distal foundation consists of iliosacral screws. The Ti6Al4V OWSER rods are inserted with the dominos placed distally (purple). The notched part of the rods (red) can only lengthen one-way through the domino’s

The implant configurations are shown radiographically in Fig. 3. All patients treated by either SDS or OWSER are allowed unrestricted physical activities postoperatively. We do not routinely perform manual axial trunk traction in the outpatient clinic for these most vulnerable of OWSER patients. For OWSER implant lengthening, we instead rely only on passive lengthening due to spinal growth, in combination with normal traction and bending generated through daily activities.

### Outcomes

The primary outcomes include coronal Cobb angle – measured on radiographs – and occurrence of SAEs (defined as unplanned medical events which (could) result in permanent disability/damage, or which requires (lengthening of) hospitalization, a re-operation, or inpatient medical managing). SAEs will be categorized as either device/surgery related (e.g. rod fracture, post-operative neurological deficit), or as disease-related (e.g. pneumonia, pain) according to a previously created complication classification system for use in EOS treatment [24]. In addition to SAEs, complications that do not meet the criteria for an SAE, but which are device/surgery related (e.g. superficial infections, proximal junctional kyphosis) will also be recorded.

Secondary radiographic measurements include parameters such as T1-T12, T1-S1 and instrumented height, T5-T12 kyphosis, L1-S1 lordosis, and pelvic obliquity. In addition, implant length will be calculated at each timepoint for both groups (SDS: Spring length, OWSER: Notched rod length) so that implant growth velocity over time can be graphed and calculated. The number of outpatient axial traction events of OWSER patients, if these are performed, will also be reported. All radiographic measurements will be performed by a trained assessor. To ensure data quality, radiographic measurements are performed semi-automatically in Surgimap v2.3.2.1 software (Nemaris, New York, USA), after which all measurements are audited by the assessor. At the end of the data collection phase, all radiographic measurements are re-assessed by a second trained assessor who is blinded to the first assessors’ measurements. Measurements are compared and disputed measurements are discussed until consensus is reached. If differences are believed to be caused by random measurement variation, the arithmetic mean of both assessors’ measurement is taken.

HRQoL will be measured with the validated Dutch version of the EOSQ-24 which consists of 24 questions in 10 domains, from which a domain- and total score is calculated [25, 26]. All outcome parameters are measured pre-operatively, immediately post-operatively, and at 1, 3, 6, and 12 months follow-up.

### Sample size calculation and statistical methods

The sample size calculation is based on potential differences between groups in maintenance of the coronal curve between the post-operative situation and 1-year follow-up. A difference in Cobb angle ≥ 5˚ is considered clinically relevant and can be reliably measured [27]. In our previous MCGR cohort studies, we found a change in Cobb angle between post-operatively and 1 year follow-up of 1˚ (SD 5.1˚) [28]. To determine differences in curve maintenance between groups ≥ 5˚ with a power (1-β) of 0.8, α = 0.05, and SD = 5.1˚, we calculated a sample size of n = 28, with a repeated measures ANOVA between factors design (comparing 2 timepoints). With an allocation ratio of 1:1, this means that 14 patients have to be included in each experimental group.

If all assumptions for performing repeated measures ANOVA are met, both systems will be compared with respect to coronal curve maintenance; age and sex will be included as covariates. For the other continuous variables, repeated measures ANOVA will be used, with the same covariates, but including all evaluated time points. If the assumptions for performing repeated measures ANOVA are not met (e.g. due to missing data), repeated measurement multilevel modelling will instead be performed, with patient, age, sex and treatment group as level 2 variables, and follow-up time as a level 1 variable.

A Kaplan–Meier survival analysis will be performed comparing both groups. The survival curves showing SAE-free survival of both groups will be statistically compared with the Log-Rank test. Analysis of EOSQ-24 scores will be performed using repeated measures ANOVA. In case there are missing EOSQ-24 item scores or questionnaires, multiple imputation with parcel summary scores will be performed using a previously published statistical method [29]. For all statistical tests, statistical significance will be set at *p* < 0.05. All statistical analyses will be performed in IBM SPSS statistics v.26.0.0.1 (Chicago, USA).

## Discussion

Current treatment of EOS is largely based on opinion and physician’s experience. This low level of evidence has many reasons, which start with the broad definition of EOS. Currently, any scoliosis that initiated before age 10 is regarded as EOS. Since many curves only become problematic during the growth spurt, these can often be treated through spinal fusion and do not require “growth-friendly” surgery. Patients that may be eligible for “growth-friendly” surgery likely had a curve onset much earlier and therefore are a distinct subgroup. Another issue is that most data comes from combined registries which include patients with heterogeneous etiologies (varying from severely disabled children to completely heathy), surgical indications and implant types. In addition, most treatment strategies are just being discovered and promising techniques such as MCGR appear much more vulnerable to failure than expected [12]. Therefore, long-term follow-up of large groups is simply absent. Finally, if results are reported, these studies are often inconsistent, incomparable, and of poor methodological quality, as reflected in a previous systematic review [30].

To investigate “growth-friendly” implants or strategies that are typically used in small numbers, we believe a prospective study on a specific population with a specific technique is essential, preferably with follow-up until after final fusion. Such a design demands a lot of effort, even before including patients, starting with a rigorous protocol, installation of a dedicated research team and IRB approval. The BiPOWR trial is the first surgical randomized trial to be performed in children with EOS, designed to investigate the limited-efficacy and safety of two novel “growth-friendly” systems.

Although the study will generate high level evidence, there are several limitations to consider. First, this study does not investigate if growth friendly surgery is actually better than continuing conservative treatment, as we only compare surgical treatments. Also, this study does not have a control group, as we compare the SDS with the OWSER, instead of a ‘gold standard’ surgical treatment – i.e., TGRs and/or MCGRs. We chose this design deliberately as we experienced many problems with current standard therapies, from which we conclude that there simply is no accepted ‘gold standard’. In fact, many of our patients are referred to our center because we have the possibility to offer these novel treatments. It would be unethical to ask these patients to be randomized to a treatment of which we know it has serious disadvantages. Obviously, the patient can always choose not to be included in the study and opt for conventional treatments such as TGR or MCGR that we perform regularly.

Second, the primary endpoint is at only one year follow-up. It is possible and likely that complications manifest after this period as is also seen with conventional “growth-friendly” systems [11, 12, 31]. The primary endpoint is, in that sense, a short-term interim analysis to look at early mechanical failures and short-term efficacy, hence the limited-efficacy nature of the study. After completion of the 1-year analysis, both cohorts of patients will be followed for several years until (and even after) skeletal maturity. Only at that point, definitive conclusions regarding efficacy can be drawn. For practical reasons, we did not incorporate the long-term analysis into the primary aim of the BiPOWR trial.

Third, although randomization is obviously a strong method to minimize bias in comparative studies, this carries the risk of being too artificial and not representative [32–34]. One way to mitigate this, is a multicenter approach and sufficient learning curve for both treatments. We recognize, however, that the investigated techniques are new, and only practiced by a selective group of surgeons. This means that the outcomes of the BiPOWR trial should only be used as an initial indication of efficacy and safety. These findings should be confirmed with larger observational studies and registry data.

## Data Availability

Access to BiPOWR study data is limited to the research team, study monitors, and the DSMB. However, the full protocol, participant-level dataset, and statistical code are available upon reasonable request after consultation with the principal investigator.

## Abbreviations

ANOVA: Analysis of Variance
BiPOWR: Bilateral Posterior One-Way Rod
CCMO: Central Committee on Research Involving Human Subjects
CE: Conformité Européenne
CoCr: Cobalt-Chromium
EOS: Early Onset Scoliosis
EOSQ-24: Early Onset Scoliosis Questionnaire
HRQoL: Health-Related Quality of Life
MCGR: Magnetically Controlled Growing Rod
OWSER: One Way Self-Expanding Rod
SAE: Serious Adverse Event
SD: Standard Deviation
SDS: Spring Distraction System
TGR: Traditional Growing Rod
Ti6Al4V: ASTM Grade 5 titanium (medical grade)
